# High-Intensity Training Increases Osteocalcin Levels: A Meta-Analysis of Effects of Exercise on Bone Remodeling biomarkers

**DOI:** 10.1186/s40798-026-00986-2

**Published:** 2026-06-02

**Authors:** Viktória Barna, Amir Makolli, Zsuzsanna Pásztorné Benyó, Marie Anne Engh, Ádám Zolcsák, Brigitta Teutsch, Vivienne Seymour, Péter Hegyi, Renáta Papp, Nora Sydo, Péter Ferdinandy

**Affiliations:** 1 Centre for Translational Medicine, Semmelweis University, Budapest, Hungary; 2 Department of Pharmacology and Pharmacotherapy, Semmelweis University, Budapest, Hungary; 3 Center for Pharmacology and Drug Research & Development, Semmelweis University, Budapest, Hungary; 4 Pharmahungary Group, Szeged, Hungary; 5 Department of Biophysics and Radiation Biology, Semmelweis University, Budapest, Hungary; 6 Department of Radiology, Medical Imaging Centre, Semmelweis University, Korányi Sándor U. 2, Budapest, 1082 Hungary; 7 Institute for Translational Medicine, Medical School, University of Pécs, Pécs, Hungary; 8 Institute of Pancreatic Diseases, Semmelweis University, Budapest, Hungary; 9 Heart and Vascular Center, Semmelweis University, Budapest, Hungary; 10 Department of Sports Medicine, Semmelweis University, Budapest, Hungary

## Abstract

**Background:**

The relationship between biomarkers of bone remodeling due to systematic training is not known, it is not possible to predict the optimal type and volume of exercise for bone remodeling.

**Objective:**

We aimed to quantify the effects of systematic training on bone remodeling biomarkers, particularly osteocalcin and bone alkaline phosphatase in healthy adult populations.

**Methods:**

This study (PROSPERO: CRD42023483811) explored the effects of systematic training (training programs lasting more than two weeks) through a systematic review and meta-analysis and included only randomized controlled trials of different types (endurance, power, and mixed) and intensities, frequency and duration of training. MEDLINE, Embase, and CENTRAL were searched on November 22, 2023, and we updated the search on 19, September 2025. Risk-of-bias and quality assessment were performed using the Cochrane Risk-of-Bias 2 (RoB2) tool and the GRADEpro Guideline Development Tool (GRADEpro).

**Results:**

A total of 16,434 records were screened, and 24 studies with 1,238 participants were analyzed. Endurance training showed no significant effect on osteocalcin level. High- and low-intensity endurance training also had no significant effect on osteocalcin. High-intensity training significantly increased osteocalcin levels (MD = 6.88; 95% CI 0.37 to 13.38). Endurance training significantly decreased the Body Mass Index (MD = −1.40; 95% CI −2.23 to –0.57). Power training showed no significant change in osteocalcin levels. Mixed training (endurance + power) did not significantly change osteocalcin levels. Bone alkaline phosphatase showed no significant change following endurance training. Bone Mineral Density showed no significant changes after endurance or power training. The risk of bias was low for all outcomes. GRADEpro assessment for osteocalcin in the high-intensity training group revealed moderate to low certainty.

**Conclusion:**

This is the first systematic review and meta-analysis on the effects of systematic training on bone biomarkers in healthy adults. Our findings show that high-intensity systematic training significantly decreases Body Mass Index and significantly increases osteocalcin levels, whereas other training types had no effect.

**Supplementary Information:**

The online version contains supplementary material available at 10.1186/s40798-026-00986-2.

## Key Points

- This meta-analysis demonstrated a significant change in the bone remodeling marker osteocalcin only in response to systematic high-intensity training.  
- Intensity, rather than the type of exercise, plays a critical role in influencing bone remodeling.
- Systematic high-intensity training significantly reduces BMI, whereas power, mixed, low or moderate-to-high intensity endurance training shows no significant effect on bone mineral density.

**Supplementary Information:**

The online version contains supplementary material available at 10.1186/s40798-026-00986-2.

## Introduction

According to the WHO Global Status Report on Physical Activity [1], 1.4 billion adults (27.5% of the adult population of the world) do not meet the recommended levels of physical activity. The increasing trend of global inactivity has a very serious impact worldwide [1]. The latest report, which analyzed trends from 2000 to 2022, found that nearly one-third of adults globally were insufficiently physically active in 2022 [2, 61]. Guidelines and recommendations emphasize that sports and physical activity strengthen muscles and bones and help prevent falls in older adults [1]. WHO recommendation suggest exercise and sport for healthy individuals at least 150–300 min of moderate-intensity activity per week [1] or alternatively 75–150 min of vigorous intensity activity per week. The American College of Sports Medicine (ACSM) recommends that adults engage in at least 150 min of moderate-intensity or 75 min of vigorous-intensity aerobic exercise per week, complemented by regular resistance, flexibility, and neuromotor training [3]. According to the ACSM guidelines, both weight-bearing aerobic activities and resistance training are important for musculoskeletal health, although the optimal type and dose of exercise for bone health remain unclear. While the benefits of training on overall bone health are well-documented [4–6], there is a gap in the knowledge focusing on the effects of different training modalities on bone health in healthy adults. The relationship between serum biomarkers of bone remodeling due to systematic training is not known, it is difficult to predict the optimal mode and volume of exercise to maximize osteogenic responses in bone and to minimize resorptive responses [7]. Circulating bone remodeling biomarkers, which reflect bone remodeling processes such as bone resorption and formation, offer a valuable tool for the real-time evaluation of bone response to training [8], but do not always imply that bone mineral balance has subsequently increased or decreased. Of these biomarkers, osteocalcin and bone-alkaline phosphatase are essential and widely used in clinical settings [9, 10]. Monitoring changes in these biomarkers can provide early insights into how different types of training affect bone health. Although circulating bone biomarkers are frequently measured in studies of bone response to exercise, results are not consistent in acute (by measuring bone biomarker responses before and after the exercise session) and systematic physical training interventions (by measuring bone biomarker responses before and after minimum 2 weeks, systematic training period). Some studies have reported an increase in biomarkers of bone resorption following training [11, 12], while others have observed an increase in markers of bone formation [13, 14]. The variability in findings is likely due to differences in study design, characteristics of participants, and the types, intensity, and duration of exercise interventions. These inconsistencies have made it difficult to draw clear conclusions about the effects of systematic training on bone biomarkers. Previous meta-analyses focused primarily on the acute effects of training [8] on bone biomarkers or concentrated on a particular group of the adult population, such as postmenopausal women [5] or obese individuals [6], and left a gap in the literature on the effects of systematic training physical activity on healthy adult populations.

The primary focus of this review was to investigate the impact of systematic training on bone-related biomarkers in healthy adults. To address this question, we applied the PICO framework (Population: healthy adults; Intervention: systematic training lasting more than two weeks; Comparator: no training; Outcome: changes in bone-related biomarkers).

## Methods

This work was carried out as part of the Systems Education Program at Semmelweis University https://doi.org/10.1038/s41591-024-03315-w and conducted within the Translational Medicine (TM) Cycle Framework by the Academia Europaea https://doi.org/10.3390/jcm9051532. The study was performed according to the Cochrane recommendations [15]. We submitted our study protocol to the International Prospective Register of Systematic Reviews (PROSPERO: CRD42023483811) and applied it accordingly (Additional File 1 Supplementary Methods S1). Overall, the study remained consistent with the pre-registered protocol. When reporting our results, we followed the guidance of the Preferred Reporting Items for Systematic Reviews and Meta-Analyses (PRISMA) 2020 Statement [16] (Additional File 2 Supplementary Table S1).

### Inclusion and Exclusion Criteria

The inclusion and exclusion criteria were applied in accordance with the PROSPERO-registered protocol. Randomized controlled trials (RCTs) comparing exercise to a comparator group without systematic training activity were eligible for inclusion. All other types of studies and animal studies were excluded. Eligible studies included healthy adults (≥ 18 years) with systematic training activities assigned and controlled by the investigators. In this study, systematic training programs were defined as training programs lasting longer than two weeks, in which participants actively engaged. For the purposes of subgroup analyses, we categorized training duration as short (≤ 16 weeks) or long (≥ 16 weeks), since systematic training beyond 3 months is more likely to elicit measurable changes in bone remodeling processes. The articles were further categorized by training type, category, frequency, and intensity. - The included studies assessed the effects of exercise on bone turnover at baseline and at the end of the intervention period. We included participants without any comorbidities, medication or supplementation, with or without systematic training or previously active lifestyle, regardless of age, sex, ethnicity, or body composition, who were compared with an untrained healthy population. The untrained population matched with the intervention group in all aspects (age, sex, ethnicity, body composition, training history, and dietary intake) except for training. Eligible studies reported changes in bone metabolism biomarkers (osteocalcin, bone-alkaline phosphatase, pyridinoline, and deoxypyridinoline) and physical parameters (body mass index and bone mineral density) compared to the untrained control group. Validated blood tests, DEXA, as well as weight and height measurement tools were used at baseline, and outcome changes were measured at the end of the studies.

### Search Strategy and Information Sources

The final systematic search was conducted on November 22, 2023, and we updated the search on September 19, 2025, without filters or restrictions in three medical databases (PubMed, Embase, and Cochrane Central Register of Controlled Trials) using the search key, detailed in Additional File 1 Supplementary Methods S2. Keywords for the search were biomarker, bone, bone-alkaline phosphatase, osteocalcin, exercise, and sport. The search syntax was slightly different to comply with the syntactic rules of the various databases.

### Data Collection

The literature search results were imported into EndNote 21 reference management software, and after duplicate removal, the selected references were imported into the Rayan AI platform [17]. The reviewers (VB, AM, VS) independently selected the records first by title and abstract, then by full text. A selection protocol prospectively written determined the whole selection process. Any disagreements during the full-text selection process were resolved by discussion. If the disagreement could not be resolved by discussion, a third reviewer (MAE) was consulted. After each selection step, Cohen’s Kappa coefficient was calculated, and only near perfect agreement was accepted (0.81–1.00). If our search did not retrieve the article on eligible studies identified in open-access libraries, we attempted to find them by contacting the authors. The references of each study included were retrieved by an online tool (citation chaser) [18] and systematically screened using the same protocol as in the original search.

### Data Items

On the basis of the pre-defined protocol, the following data were extracted from both intervention and comparator groups: study characteristics, population description, intervention characteristics, and outcome parameters. We collected the mean or median of the outcome data. The outcome measures were changes in osteocalcin, bone alkaline phosphatase, pyridinoline, and deoxypyridinoline compared to pre-treatment conditions, assessed immediately after intervention and at follow-up, if applicable, via blood serum or urine analysis. We also collected physical parameters such as body mass index and bone mineral density (assessed by DEXA, in-body measurements, or medical imaging methods). Exclusion criteria rejected outcomes beyond bone metabolism biomarkers and bone density/body mass index changes. If data were only available in graphical form, we performed the extraction with GetData Graph Digitizer software (v.4.6.) [19].

### Synthesis Methods

Eligible studies were prepared for data extraction by systematic categorization (Table 1). The eligible articles were first categorized by outcome type (endurance, power, and mixed training). During the extraction process, we sub-grouped the articles by duration, frequency and intensity (Additional file 1 Supplementary Methods S3). As these differences were related to training characteristics, the eligible studies were systematically categorized for synthesis. Outcome changes were analyzed according to intervention or control status within these groups.

**Table 1:** The categorization of different training types

Training type	Training category	Frequency	Intensity	Duration
Endurance	Aerobic/Anaerobic	High frequency (≥ 3 times/week)/Low frequency (≤ 3 times/week)	Low intensity (≤ 70% HR max)/ Moderate-to-high intensity (70–85% HR max) High intensity (≥ 85% HR max)	Short duration (≤ 16 weeks) Long duration (≥ 16 weeks)
Power	Strength training	High frequency (≥ 3 times/week) Low frequency (≤ 3 times/week)	Low intensity (≤ 80% of 1-RM) Highintensity (≥ 80% 1-RM)	Short duration (≤ 16 weeks) Long duration (≥ 16 weeks)
Mixed	Endurance	High frequency (≥ 3 times/week) Low frequency (≤ 3 times/week)	Low intensity (≤ 70% HR max) Moderate-to-high intensity (70–85% HR max) Highintensity (≥ 85% HR max)	Short duration (≤ 16 weeks) Long duration (≥ 16 weeks)
	Low-intensity power training Moderate-to-high intensity power training	High frequency (≥ 3 times/week) Low frequency (≤ 3 times/week)	Low intensity (≤ 80% of 1-RM) Highintensity (≥ 80% 1-RM)	Short duration (≤ 16 weeks) Long duration, (≥ 16 weeks)

Categorization of the eligible articles by training characteristics based on the 2020 ESC Guidelines, European Federation of Sports Medicine Associations (EFSMA) exercise prescription of health (EPH) the definition of FITT and the American College of Sports Medicine. *HR max* maximum heart rate, *1-RM* One-Repetition Maximum. The categorization process for training type, category, frequency, intensity was based on the training classification by Mitchell et al. [57] (power, endurance, and mixed), ESC guidelines [58] (endurance vs. anaerobic endurance exercise) and European Federation of Sports Medicine Associations (EFSMA) exercise prescription of health (EPH) [59] (moderate-to-high, low-intensity, and high-intensity sports) and the American College of Sports Medicine [3]

The 2020 ESC Guidelines on Sports Cardiology and Exercise in Patients with Cardiovascular Disease define vigorous (high) intensity exercise as ≥ 70% of HR max in healthy adults. The European Federation of Sports Medicine Associations (EFSMA) Exercise Prescription for Health consensus specifies vigorous intensity as 65–85% of HR max. In addition, the ACSM guidelines distinguish five intensity zones, which partly overlap with both the ESC and EFSMA recommendations. Since our analysis focused on preventive and health-promoting aspects in healthy populations, we defined moderate-to-high intensity as 70–85% of maximal heart rate (HRmax). Studies reporting training intensities ≥ 85% HRmax, were included in the high-intensity group. To ensure consistency across all three guidelines, we therefore categorized training intensity into three groups: low, moderate-to-high, and high intensity.

### Statistical Methods

In medical meta-analyses, the use of a random-effects model was crucial to accommodate the variability among included studies, considering their different settings, and to obtain meaningful pooled effect sizes. For continuous variables, the mean difference (MD) was calculated with a 95% Confidence Interval (CI). For bone alkaline phosphatase we calculated the effect size as Hedges’g standardized mean difference (SMD) with 95% CI [20]. As QoL results were presented at various points in the articles, a multivariate model was employed. The findings of this meta-analysis were summarized in forest plots. Where applicable (the number of studies was large enough and studies were not too heterogeneous), prediction intervals for the results were also reported. In addition, between-study heterogeneity was assessed using both Higgins and Thompson's I^2^ statistics [15] and tau (T2), when applicable (e.g. when more than five studies were included). The effect of potential outlier publications was explored using different measures of influence and plots as recommended by Harrer et al. [21]. Results were considered statistically significant if the pooled CI did not contain null value. All statistical analyses were performed by R [22] using the meta package for basic meta-analysis calculations and plots, and the dmetar [23] package for additional influential analysis calculations and plots. The metafor package [24] was also used for multivariate models.

### Risk of Bias Assessment and Aggregated Evidence Synthesis

A risk-of-bias assessment was performed with the revised Cochrane risk-of-bias tool (RoB2) [25] by two independent authors (VB, and VS). Discrepancy checks between the two authors were resolved by consensus.

### Quality Assessment of Included Studies

Confidence in the quality of evidence for the meta-analysis was assessed using GRADEpro [26] through the Grade Pro program by two independent authors (VB, VS). Disagreements were resolved by consensus.

## Results

### Literature Search, Study Selection and Study Characteristics

**Fig. 1:**
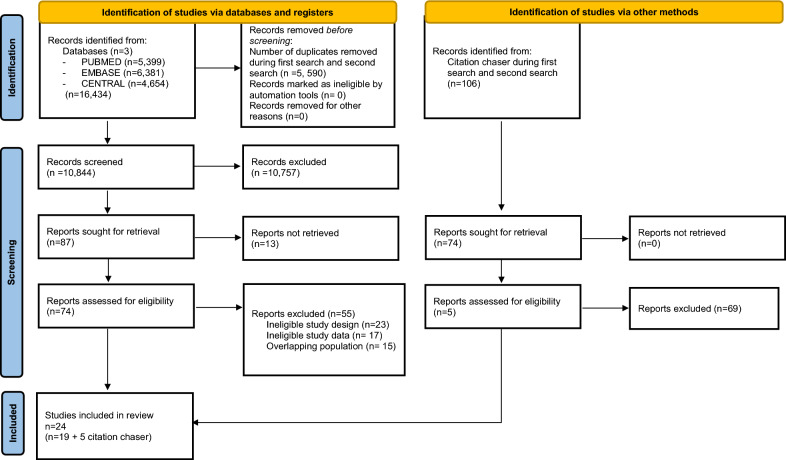
PRISMA flowchart of the selection process

The results of the literature search and study selection processes are summarized in Fig. 1. We identified 16 434 records; after duplicate removal, 10, 844 were selected, and 74 were eligible for full-text screening. Native speakers helped to translate non-English papers [7] into English. We sent requests for additional information to authors of studies that met the selection criteria, but reported confusing data or measurement techniques. One author provided supplementary material and we included the article into the analysis [27]. One author could not provide us with an adequate explanation of the data; therefore, we excluded that article [7]. Due to the lack of response [14], we used the data reported from the article, but calculated standardized mean difference (SMD) in the analysis to pool the results. For bone remodeling biomarkers in the articles screened, we found sufficient data for only osteocalcin and bone-alkaline-phosphatase to include in the quantitative analysis. More data would be needed to include pyridinoline and deoxypyridinoline in the quantitative analysis. Thirteen reports could not be retrieved because ten articles were duplicates, one report was an RCT protocol, one was an abstract of a future trial, and another one was only a conference paper. These thirteen reports were not included in the analysis because we could not extract data from them, nor could we find the articles published. After full-text selection, 19 articles were eligible for data extraction. We identified five new eligible items [28] during the citation chaser phase and a total of 24 articles were included**.** One article was not included in the quantitative analysis [28], because the result was reported as a median, while all other articles included reported results as means. In the mixed training type subgroup, the value of 1-RM was not defined in all cases because this was not possible due to the kind of training [29]. In this study, participants performed endurance exercise with low weight, where the 1-RM was not relevant. In one study [30], the second study arm started the exercise after 6 months of initial inactivity; for statistical reasons, only the first 6 months of the study were included in the analysis. One study conducted super jumping on a trampoline, categorized as resistance training due to high muscle activity and low heart rate. One study analyzed only the non-supplemented group [31]. Baseline characteristics of the studies included are shown in Table 2.

**Table 2:** Baseline characteristics of the studies included in the quantitative analysis

Reference	Population (healthy adults)			Intervention (systematic training)			Comparator	Outcome
	Study location	Total number of participants (intervention/control) and female %	Age (mean SD)	Training type/frequency	Training intensity	Training duration	Age (mean SD)	
Alev et al. [46]	Turkey	50 (25/25) 100%	47 ± 7	Endurance/high frequency	High intensity	Short duration	49 ± 5	BAP
Anek et al. [29]	Thailand	60 (44/16) 100%	EN:39.86 ± 3.7 M:40.92 ± 4 M:40.26 ± 3.2	Mixed/high frequency	Moderate-to high intensity	Long duration	41.25 ± 3.3	OC BMI
Carneiro et al. [11]	Portugal	54 (40/14) 100%	LF: 67 ± 5 LF:67 ± 3 HF:68 ± 4	Endurance/high frequency	High intensity	Long duration	67.5 ± 5	OC, BMD
Danz et al. [30]	Germany	83 (46/37) 100%	51.9 ± 4.5	Endurance/high frequency	Moderate-to high intensity	Long duration	51.5 ± 4.1	OC
Helge et al. [37]	Denmark	26 (18/8) 0%	EN: 68.0 ± 4 P: 69.1 ± 3.1	Endurance Power training/ high frequency	High intensity Low intensity power	Long duration	67.4 ± 2.7	OC BMD
Rodziewicz-Flis et al. [31]	Poland	37 (9/9) 100%	72.9 ± 5.3	Mixed/high frequency	Moderate-to high intensity	Short duration	72.9 ± 5.3	OC
Guadalupe-Grau et al. [39]	Spain	66 (28/38) 35%	Male:23.9 ± 2.4 Female:23.2 ± 2.7	Mixed/high frequency	Low intensity endurance + high intensity power training	Short duration	Male:23.13 ± 2.2 Female: 23.7 ± 2.7	OC, BMD
Hornstrup et al. [12]	Denmark	26 (14/12) 0%	24.2 ± 2.8	Endurance/high frequency	High intensity	Short duration	25.8 ± 2.8	BMD, OC
Kortas et al. [33]	Poland	36 (18/18) 100%	66.78 ± 4.76	Endurance/high frequency	Moderate-to high intensity	Short duration	66.12 ± 4.83	OC
Grove et al. [40]	USA	15 (10/5) 100%	LI:56 ± 4 HI: 54 ± 1.9	Endurance/high frequency	Low and moderate to high intensity	Long duration	56 ± 4.5	BMD
Kim et al. [32]	Korea	39 (29/10) 0%	24.86 ± 2.80	Endurance/high frequency	Moderate-to high intensity	Short duration	26.6 ± 2.8	BMI, BMD, OC, BAP
Kishimoto et al. [43]	USA	26 (13/13) 100%	20.8 ± 1.1	Power/high frequency	Low intensity	Short duration	20.4 ± 1.4	OC
Hatori et al. [14]	Japan	35 (23/12) 100%	hi1:58 ± 5 hi2:56 ± 4	Endurance/high frequency	High intensity	Long duration	58 ± 8	OC, BAP
Pereira et al. [35]	Portugal	67 (41/26) 100%	67.3 ± 6.5	Endurance/high frequency	High intensity	Long duration	69.9 ± 5.4	OC
Remes et al. [13]	Finland	140 (70/70) 0%	57.6 ± 2.96	Endurance/high frequency	Low intensity	Long duration	57.8 ± 2.8	OC
Sanchez-Trigo et al. [27]	Spain	42 (24/18) 100%	40.83 ± 4.4	Endurance/high frequency	Low intensity	Short duration	42.22 ± 5.69	OC
Schroeder et al. [44]	USA	37 (28/9) 100%	li:24.4 ± 1.9 hi:24 ± 1.4	Power /low frequency	Low and high intensity	Long duration	24.4 ± 2.2	OC
Vainionpää et al. [60]	Finland	76 (37/39) 100%	38.1 ± 1.9	Endurance/high frequency	Low intensity	Long duration	38.2 ± 1.6	BMD
Vasto et al. [42]	Italy	24 (12/12) 100%	18–40	Power/high frequency	Moderate-to high intensity	Long duration	18–40	OC
Vincent et al. [45]	Usa	62 (46/16) na	li 67.66 hi: 66.6 ± 7	Power/high frequency	Low and high intensity	Long duration	71 ± 5	BMD, OC
Guzell et al. [34]	United Kingdom	24 (12/12) 100%	55.67 ± 3.44	Endurance/high frequency	Moderate-to-high	Short duration	54.42 ± 4.01	BMI
Osalou et al. [41]	Turkey	24 (14/10) 100%	36.10 ± 3.38	Endurance/high frequency	High intensity	Short duration	36.10 ± 3.38	BMI, OC, BALP
Yaris et al. [38]	Not given	32 (15/17) 100%	53.78 ± 3.93	Endurance/high frequency	High intensity	Short duration	54.54 ± 5.52	BMI, OC

*BAP* Bone-alkaline Phosphatase, *OC* Osteocalcin, *BMD* Bone Mineral Density, *BMI* Body Mass Index, *EN* Endurance training study arm, *M* Mixed Training Study arm, *LF* Low Frequency Study arm, *HF* High Frequency Study arm, *P* Power training study arm, *LI* Low Intensity study arm, *HI* High Intensity study arm, *HR Max* Maximum Heart Rate

### Quantitative Analysis of the Effect of Systematic Endurance Training on Osteocalcin Levels

Of bone remodeling biomarkers, only osteocalcin and bone-alkaline-phosphatase were analyzed A total of 14 studies (607 patients) were pooled to assess the effect of endurance training on osteocalcin levels. The overall statistical analysis (Additional File 3 Supplementary Material Figure S1) showed a non-significant mean difference (MD:2,24;95% CI -0,72to5,21;*I*^*2*^ = 82%). We found outliers after the analysis,therefore, we performed a subgroup analysis based on training intensity (moderate to high, high-intensity and low-intensity).

### Subgroup Analyses

#### Effect of Moderate-to-High Intensity Endurance Training on Osteocalcin Levels

**Fig. 2:**
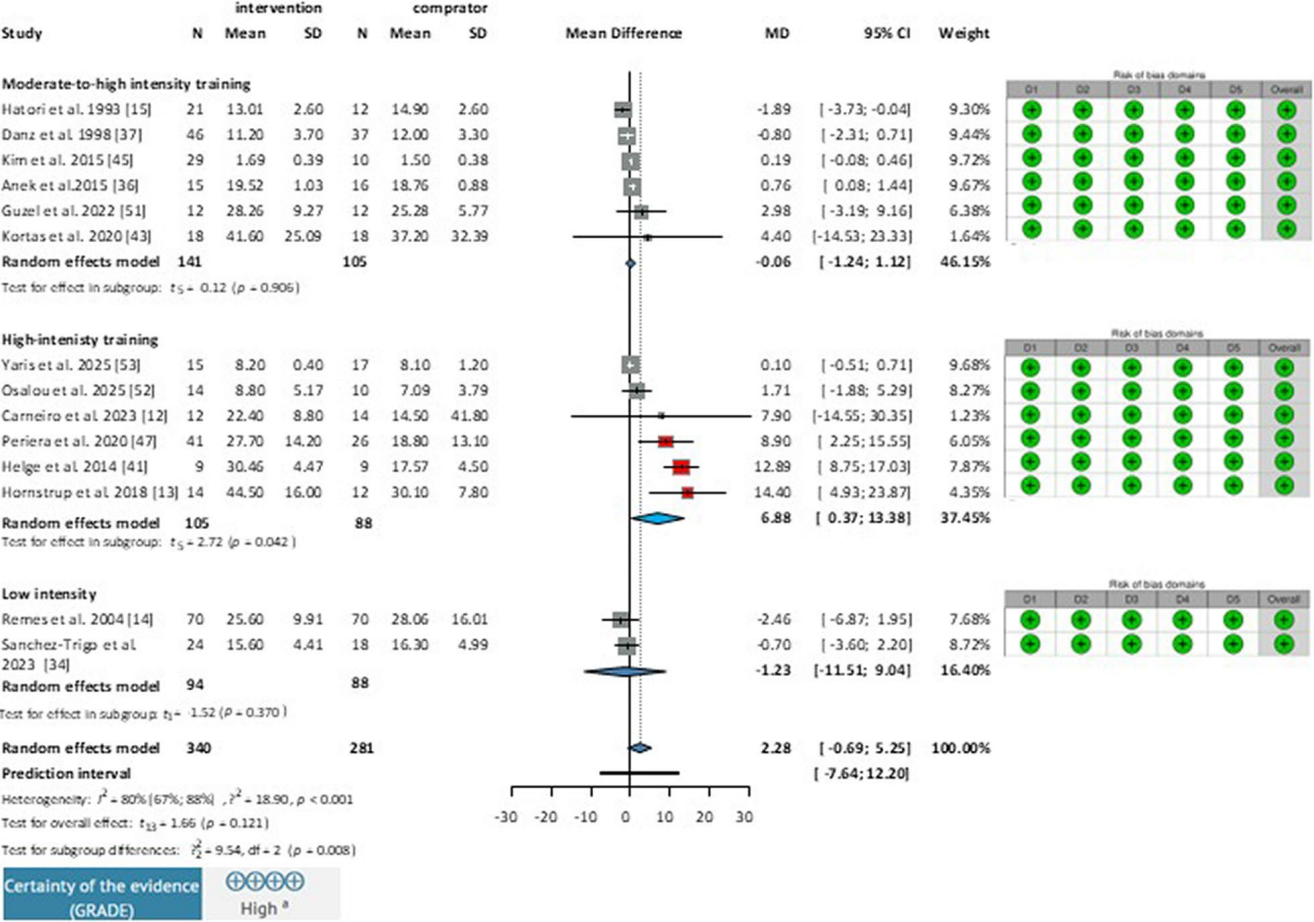
Effect of endurance training on osteocalcin levels in healthy adults*. MD* mean difference, *CI* Confidence Interval; *D1* Bias due to randomization process, *D2* Bias due to deviations from intervention intended, *D3* Bias due to missing outcome data, *D4* Bias in measurement of the outcome, *D5* Bias in selection of the results reported.

The moderate-to-high group included 6 studies with a total of 246 participants (Fig. 2). Training intensity was 75–85% of the maximum heart rate in all studies, with workout sessions lasting 40–60 min each and at least three sessions per week (high frequency). Types of training included running [32] and aerobic step exercise [29] and moderate-to-high intensity walking [14, 33]. Three studies were of long duration, lasting more than 16 weeks [14, 29, 30], while three were of short duration, lasting less than 16 weeks [32–34]. There were five articles on women [14, 29, 30, 33, 34], and one on men [32]. One study [14] investigated two experimental arms, and these arms were merged for analysis, as the type of training (moderate-to-high intensity walking) was the same in both, differing only in the specific moderate-to-high ranges. In one study [29] multiple interventions were studied, but in this subgroup, only the step endurance (only endurance training) training group was analyzed. The analysis of the moderate-to-high intenisty training subgroup showed a non-significant mean difference ( % (MD = −0.06; 95% CI −1.24 to 1.12; I^2^= 80%) . 

#### Effect of High-Intensity Endurance Training on Osteocalcin Levels

Six articles, with 193 participants, were included in the high intensity training group (Fig. 2). The studies in the high-intensity group were classified as such because the continuous exercise load was maintained above 85% of HR max throughout the sessions. Training sessions lasted 50–60 min each, 1–3 times per week. Exercise types included recreational team handball [11, 35, 36] and football [12, 37]. One study investigated the effect of HIIT (high-intensity interval) stationary bike exercise [38]. In one study [11], there were three intervention arms with low (1–2 sessions per week) and high (3 sessions per week) training frequencies, conducted over long periods of time (more than 16 weeks). Another study [37] had two arms, comparing the effect of resistance training to handball; therefore, we only included the endurance training arm in this analysis. The study population consisted of middle-to-older aged males [11, 37], young untrained men [12] and postmenopausal women [35, 38]. The statistical analysis of the training subgroup showed significant mean difference (MD:6,88;95%CI0.37to13.38; I^2^=80%).

#### Effect of Low-Intensity Endurance Training on Osteocalcin Levels

Two articles were included in this group (Fig. 2), with a total number of 182 participants, where training intensity was under 70% of the maximum heart rate, with sessions lasting 40–60 min each, and with a high frequency of at least three sessions per week. The studies investigated the effects of walking in premenopausal women [27] and the effects of brisk walking in middle-aged men (age of 57.6 ± 2.9) [13] for a long period of more than 16 weeks. The statistical analysis of the low-intensity training subgroup showed a non-significant mean difference (MD = −1.23; 95% CI −11.51 to 9.04; I^2^= 80%) (.  

#### Effect of Long-Duration Endurance Training on Osteocalcin Levels

We performed a subgroup analysis based on training duration (see Additional File 3 Supplementary Material Figure S2). Due to lack of studies, only osteocalcin was analyzed. The method of stratification was based on the length of training (short- and long-duration subgroups). In the long-duration subgroup, a total of 7 studies were included, involving a total of 386 patients*.* The duration of the training was longer than 16 weeks. The population included consisted of females performing moderate-to-high-intensity training [30, 37] men performing low intensity training [13] and males and females performing high intensity training [11, 35, 39]. The statistical analysis of the long-duration training subgroup showed a non-significant mean difference (MD: 2.82; 95%CI2.86to8.51; *I*^*2*^= 65%). 

#### Effect of Short-Duration Endurance Training on Osteocalcin Levels

A total of 7 studies were included in the short-duration subgroup involving 223 patients (see Additional File 3 Supplementary Material Figure S2). The duration of the training was less than 16 weeks. The population included females low [27] moderate-to-high [34, 40] and high intensity training group [12, 38, 41] and males in moderate-to-high intensity group [32].The statistical analysis of the short-duration training subgroup showed a non-significant mean difference (MD: 0.19;95% 95 % CI−0.22 to 0.61; *I*^*I*^ I^2^ 87%). t   

#### Effect of High-Frequency Endurance Training on Osteocalcin Levels

We performed a subgroup analysis based on training frequency (see Additional File 3 Supplementary Material Figure S3), including only high-frequency training studies (more than 3 times per week). The 14 studies that we included in the analysis of the endurance training group belonged to the high-frequency training group. The overall statistical analysis of the high frequency subgroup showed a non-significant mean difference(MD: 2.32;95%CI0.64to5.27; *I*^*2*^= 79%). t 

#### Effect of Power Training on Osteocalcin Levels

Five articles, with a total of 131 participants, examined the effect of power training on osteocalcin [39, 42–45]. Data were sufficient for power training for a quantitative analysis of changes in osteocalcin levels. In one case [33], we analyzed the experimental arm where power training was investigated. The studies involved elderly men [33, 45], young women [44], middle-aged females [42] and female college students [43] with training periods ranging from 2 weeks to 12 months. Training types included power [37], progressive resistance power [44, 45] and plyometric exercises [42, 43] with varying frequencies (2–5 times per week) and intensities (50–125% of 1RM or impact forces ≥ 3 × body weight). The overall statistical analysis of the power training group showed a non-significant mean difference (MD: 3.05; 95%CI −0.72 to 6.81;*I*^*2*^ = 61%) (Additional File 3 Supplementary Material Figure S4). The analysis of the effects of high intensity power training (Additional File 3 Supplementary Material Figure S5) on osteocalcin levels did not show significant differences (MD: 3.73;95% −0.59 to 8.06; *I*_*2*_= 72%).

#### Effect of Mixed Training on Osteocalcin Levels

We had sufficient data for mixed training to quantitatively analyze only changes in osteocalcin levels, as we found only found one article [45] on bone-alkaline phosphatase. The subgroup analysis of the effect of mixed training included 3 studies with 127 participants (Additional File 3 Supplementary Material Figure S6). Due to the small number of studies available, further subgroup analysis was not possible. The studies examined middle-aged [37] and elderly women [31], young males and females [39], with training periods ranging from 9 to 16 weeks. Training types included moderate-intensity endurance and resistance exercises [31, 37] and higher-intensity power training mixed with plyometric jumps [39] at a frequency of 3 days per week. For multiple study arms, only the mixed training study arm [37] was included in this group. For supplementations [31], only the group that did not receive vitamin D supplementation alongside training was analyzed. The overall statistical analysis of the group showed a non-significant mean difference (MD: 0.9; 95%CI −3.28 to 5.08; *I*^*2*^=6% (

#### Effect of Endurance Training Bone Alkaline Phosphatase Levels

**Fig. 3:**
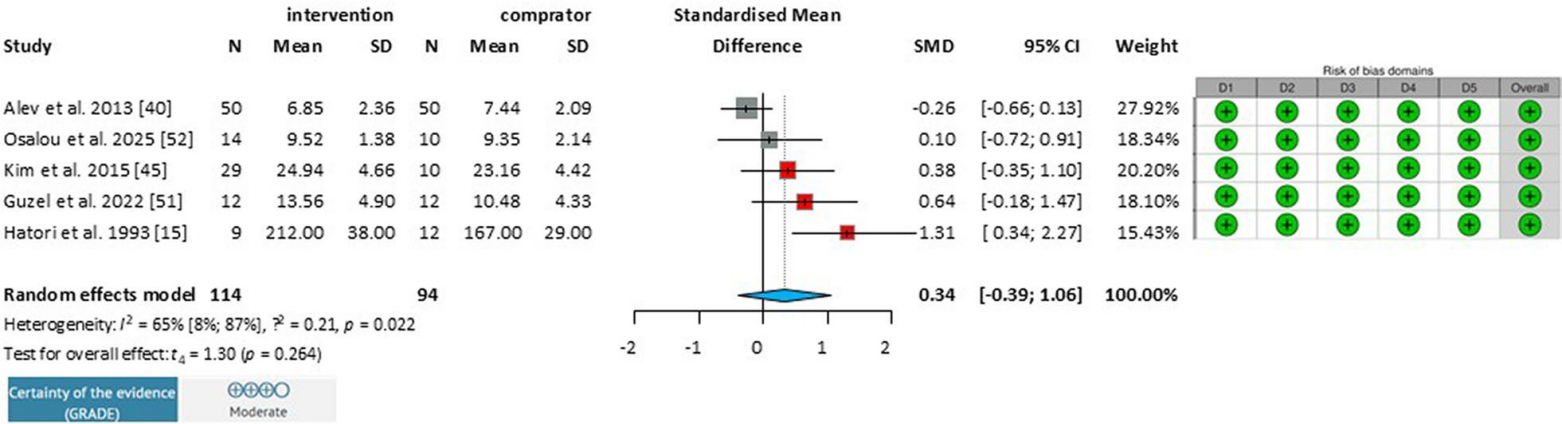
Effect of endurance training on bone-alkaline phosphatase level in healthy adults. *MD* mean difference, *CI* Confidence Interval, *D1* Bias due to randomization process, *D2* Bias due to deviations from intervention intended, *D3* Bias due to missing outcome data *D4* Bias in measurement of the outcome, *D5* Bias in selection of the reported results

A total of 5 studies were selected for analysis, involving a total of 208 patients (Fig. 3)*.* Due to the small number of studies available and differences in training characteristics, further subgroup analysis of bone alkaline phosphatase was not possible. Training duration was shorter [32, 41] or longer [14, 34, 42] than 16 weeks. The population included consisted of postmenopausal females who trained at low or high intensity [14, 34, 37, 41] and young males who trained at high intensity [32]. The overall statistical analysis of the group showed a non-significant mean difference (MD: 0.34; 95% CI −0.39 to 1.06; *I*^*2*^ = 65%).

#### Effect of Endurance Training on Body Mass Index (BMI)

**Fig. 4:**
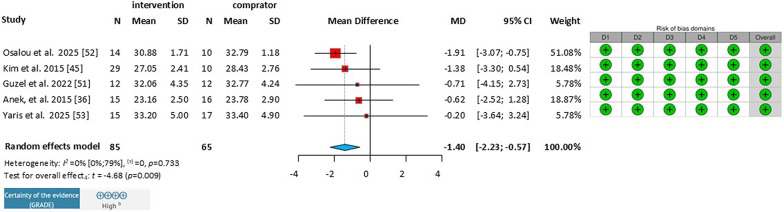
Effect of endurance training on Body Mass Index in healthy adults. *MD* mean difference, *CI* Confidence Interval, *D1* Bias due to randomization process, *D2* Bias due to deviations from intervention intended, *D3* Bias due to missing outcome data, *D4* Bias in measurement of the outcome, *D5* Bias in selection of the reported results

Among the articles analyzed, we found five that also reported both pre- and post-intervention BMI. All five were selected for analysis, involving a total of 150 patients (Fig. 4).

These studies included moderate-to-high intensity mixed training for working women [46], low-intensity walking for postmenopausal sedentary women [34], moderate-to-high endurance exercise for obese men [32] and women [41] and high intensity interval training on stationary bike [38]. The duration of the training ranged from 8 to 16 weeks, with 3–4 sessions per week.

The overall statistical analysis of the group showed a significant mean difference (MD: −1.40;95% CI: −2.23 to 0.57;*I*^*2*^ = 0%).

#### Effect of Endurance Training on Bone Mineral Density (BMD)

As a secondary outcome, six studies reporting data on BMD were also included in the overall analysis (Additional File 3 Supplementary Material Figure S7).The studies included premenopausal women who performed jumping [42], elderly men (high intensity recreational football and handball) [11, 12, 39] and young men [32] (moderate-to-high intensity running 4 times per week). The duration of the training ranged from 8 weeks to 12 months. Two subgroups were created from the studies, high-frequency (training sessions more than 3 times per week) and high-intensity.

#### Effect of High-Frequency Endurance Training on BMD

In the high-frequency (3 or more than 3 times per week) subgroup, a total of 2 studies were selected for analysis, involving a total of 119 patients (Additional File 3 Supplementary Material Figure S8). The statistical analysis of the high-frequency training subgroup showed no effect on the mean difference (MD: 0.00;95%CI−0.12 to 0.12;*I*^*2*^ = 0). 

#### Effect of High Intensity Endurance Training on BMD

In the subgroup, a total of 4 studies were selected for analysis involving a total of 76 patients (Additional File 3 Supplementary Material Figure S8). The statistical analysis of the moderate-to-high training subgroup showed no effect on the mean difference (MD: 0.00;95%CI −0.12 to 0.11;*I*^*2*^ = 33%). 

#### Quality Assessment of Included Studies

The assessment of osteocalcin in all subgroups resulted in high certainty. In terms of BMI certainly, the assessment produced high certainty. Additional File 2 Supplementary Table S2 shows the results of the assessment.

#### Risk of Bias Assessment

A comprehensive explanation of this assessment is provided in Additional File 2: Supplementary Table S3. The intervention groups from each selected study were evaluated according to outcomes and the weight of statistical analysis (assessment ID, outcome type, intervention type). For all articles included in the meta-analysis, the overall risk of bias assessed by RoB2 was low. Additional File 2 Supplementary Table S3 shows the results of the assessment.

## Discussion

This study is the first systematic review and meta-analysis to summarize the results of randomized controlled trials currently available, investigating the effects of systematic training on bone biomarkers in healthy adults, specifically osteocalcin and bone-alkaline phosphatase. Our study demonstrates that only systematic high intensity training affects osteocalcin levels while moderate-to-high and high-intensity endurance training significantly decreased BMI. Other exercise types such as low-intensity, power, and mixed training show no effect on osteocalcin, bone-alkaline phosphatase, body mass index and BMI. Our findings highlight that where the synergistic effects of high intensity, dynamic loading, and rest periods were simultaneously present during training, significant differences in bone metabolism markers were observed. After analyzing training effectiveness of different training frequencies and duration, we also found that changes in bone biomarkers were not dependent on duration or frequency, but rather on intensity.

### Effect of Moderate to High Intensity Endurance Training on Osteocalcin Levels

The overall analysis of the subgroup showed no significant changes in osteocalcin levels. However, some individual studies showed significant differences in the groups. Significant increases in osteocalcin levels were higher in middle-aged women in the group using dumbbell weights than those without [29]. The change in osteocalcin levels was more pronounced in semi-and postmenopausal women and occurred sooner in the group that used wrist weights during their exercise sessions [30]. Continuous walking, even at high intensity (above the anaerobic endurance threshold), did not show significant changes in osteocalcin levels in postmenopausal women [14], while Nordic walking training induced a significant increase in the osteocalcin levels in elderly women, when training was mixed with power training in the second micro-cycle during the training period [33]. High intensity running among young obese men showed [32] changes in osteocalcin after exercise.

Based on individual study results, when high-intensity training was accompanied by other stimulus, such as fast muscle contractions with light wrist weights during endurance training or high BMI, it not only elicited a higher heart rate response, but also affected bone metabolism. The reason behind this is most likely that high-intensity dominant endurance training, accompanied by fast muscle contractions with low weight, lead to a greater magnitude of loading forces [47], and this stimulates bone metabolism more effectively than a general endurance exercise program [29].

### Effect of High-Intensity Endurance Training on Osteocalcin Levels

The increase in osteocalcin levels was higher in middle-aged and older males than the increase in bone resorption markers, resulting in a positive net bone remodeling balance after even one 60-min session per week [11]. Elderly men who played recreational football 2–3 times a week for 16 weeks also showed an increase in osteocalcin levels [37], which was consistent with the results of other studies included. This intervention is particularly interesting because this is the first study to investigate the osteogenic effect of recreational football in comparison with resistance training in elderly men. In this study, bone resorption was similar after football and resistance training, indicating that the osteogenic impact of football was primarily due to its effect on bone formation, leading to a positive bone balance and an increase in bone mineral density. In contrast to recreational football, resistance training did not seem to have any osteogenic effects, either after 4 or 12 months. A possible explanation is that the resistance training was too low intensity to stimulate bone remodeling [37] and football is a contact sport that also has a stimulating effect on bone remodeling. A study with younger men (recreational team handball for 12 weeks) also showed similar increases in osteocalcin levels [12]. This means that by performing high intensity training for 16-weeks [37], middle-aged and older males were able to increase bone remodeling markers to the same extent as younger populations. After 16 weeks, recreational team handball training for postmenopausal women resulted in an increase of osteocalcin levels, and an overall osteogenic change in bone remodeling [35].

Our results show that compared to the endurance dominated training with low weight in the high-intensity group, the change in osteocalcin levels was significant even without weight training in the high-intensity group. A possible explanation for the similar effect of high-intensity training in heterogeneous populations may be that high-intensity trainings and osteogenesis are upregulated by high-impact forces [48]. During high-intensity training three factors exist at the same time: elevated heart rate, dynamic movement in the form of specific high-intensity actions such as stops, jumps, changes of direction and speed, and short rests between high-intensity sessions [11]. These factors have significant potential to positively influence bone remodeling, highlighting the osteogenic effects of in various populations [49]. Substantial increases in osteocalcin levels after a seven-week high-intensity training protocol were observed in another study on intermittent running [49]. The acute effects of high-intensity training on osteocalcin were further demonstrated [50], where osteocalcin showed transient increases after high-intensity training sessions. The dynamic relationship between and osteocalcin forms were highlighted earlier [51], where transient increases were observed in total osteocalcin and undercarboxylated osteocalcin after high-intensity training sessions. A previous meta-analysis [52] also concluded that high-intensity training may be a more efficacious intervention than other traininginterventions or no-intervention controls in older adults, but further investigation in this area may help optimize protocols. Previously, acute effects of high-intensity training on osteocalcin levels were also demonstrated [50] where osteocalcin showed transient increases during high intensity training sessions. Animal studies found that the fluctuation of lactate during high-intensity training may be an important co-factor in stimulating bone metabolism, but this hypothesis has not been tested in a human population [53].

Our results show that high-intensity systematic training not only increases both endurance and endurance capacity but also affects bone metabolism more effectively than traditional endurance exercises. This may be due to differences between exercise characteristics, but the underlying mechanism during high-intensity training needs further investigation.

### Effect of Low-Intensity Endurance Training on Osteocalcin Levels

Low volume walking improved cardiometabolic health and body composition without affecting bone remodeling markers in postmenopausal women [34]. In low-intensity training groups, daily walking mixed with low-impact jumping [27] did not affect osteocalcin or bone remodeling markers, and prolonged low-intensity exercise over 4 four years had no significant impact on osteocalcin [13]. This is because low-intensity activities do not sufficiently stimulate osteocalcin-related bone formation pathways [27] and do not induce substantial changes in bone remodeling or osteocalcin levels [13] due to the lack of training-related osteogenic factors [49].

### Effect of Power Training on Osteocalcin Levels

We observed outlier values that showed greater changes in osteocalcin levels in high-intensity resistance training studies where short rest periods were allowed [42, 44, 45], while continuous moderate resistance training and single jump training [43] showed an improving effect, but the overall difference was not significant. Differences in osteocalcin responses may be explained by the positive effect of resistance exercise on osteocalcin levels in cases where high intensity power training was performed. Higher resistance loads (e.g., 80% of 1RM) were associated with greater osteogenic responses and elevated osteocalcin response compared to low-intensity protocols [45]. In contrast, continuous resistance training without dynamic loading and high intensity did not show a significant improvement [29].

Our findings are in alignment with a previous meta-analysis [54] that focused on bone health and osteocalcin levels in postmenopausal women. This meta-analysis confirms the benefits of resistance training on the physical fitness of postmenopausal women, although there is more debate on its influence on bone metabolism.

### Effect of Mixed Training on Osteocalcin Levels

We also observed the same patterns in a subgroup analysis of the effect of mixed training on osteocalcin levels. The greatest change in osteocalcin level was observed in the study [39] with structured rest, moderate-to-high efforts and greater cumulative mechanical and dynamic loading. The result was the same, but less meaningful in continuous, moderate-to-high mixed training, without rest periods or dynamic loading in power group [29]. During progressive training, where intensity was high, but only at the end of the study, we observed less change in osteocalcin levels [31]. Despite the positive results of the individual studies, the overall change in osteocalcin levels in the mixed-training group was not significant, due to the high heterogeneity of the studies (differences in training load, rest periods, intensity, and dynamic loading), although combining moderate-to-high endurance and resistance exercises modify osteocalcin levels more than either modality alone, suggesting that complex interventions has synergistic benefits on bone remodeling [7].

### Effect of Endurance Training on Bone Alkaline Phosphatase Levels

The other bone metabolism biomarker, bone alkaline phosphatase, was analyzed from four studies. Studies with higher-intensity protocols [14, 32] showed greater, although non-significant increases in bone-alkaline phosphatase compared to low-intensity protocols [34, 46]. Although some studies showed significant differences, the overall change in bone alkaline phosphatase was not significant. This is because, although low-intensity training can maintain bone health, it does not increase bone mass, as it lacks the necessary intensity and dynamic movements to effectively stimulate bone formation [55].

### Effect of Training on BMI and BMD

Moderate-to-high continuous running and endurance training showed a significant reduction in Body Mass Index and low-intensity continuous endurance training improved body composition without affecting bone mineral density [34]. Low-intensity or moderate-intensity training, such as walking or moderate-endurance exercises, did not produce significant changes in BMD unless mixed with weight load [32, 40]. High intensity such as recreational football and handball showed improvements in BMD without weight-bearing [11, 35–37]. The reason for this mechanism is that moderate-intensity endurance exercise is beneficial for cardiometabolic health but does not generate sufficient skeletal strain to stimulate significant changes in BMD [32]. Positive changes in BMD occur when weight-bearing or dynamic, impact-based activities are included, as these generate the necessary power of stress on the skeleton needed for bone remodeling [11, 35–37]. To stimulate the osteogenic effects for bone mass accretion, bone tissues must be exposed to mechanical load, intensity and dynamic movement during activities [56].

### Strengths and Limitations of the Study

This is the first meta-analysis to investigate the effects of systematic training on healthy adults, comparing the effects of different training modalities (endurance, mixed, and power). We identified the benefits of the synergistic effects of training in healthy adults without comorbidities or supplementation to understand how training affects bone biomarkers in the absence of confounding factors. The studies included were of high quality. However, there are some limitations to the study such as small sample sizes, lack of long-term data on bone mineral density, heterogeneity of studies, particularly in training modalities, intensity, duration, and participant characteristics, which limited the pooling of the results. The effects of systematic training were evaluated independently of participants’ baseline activity level or prior training status. We acknowledge that participants with different baseline characteristics and training backgrounds were included, which may have influenced the observed outcomes. Osteocalcin and bone-alkaline phosphatase are valuable markers of bone remodeling, but do not fully describe the complexity of the whole bone remodeling process. Since there is currently no guideline that distinguishes between short- and long-term training programs, we categorized training duration as short (≤ 16 weeks) or long (≥ 16 weeks), as systematic training beyond 3 months is more likely to elicit robust changes in bone remodeling processes. Studies included in this meta-analysis reported that participants maintained their habitual diet without any prescribed caloric restriction. Nevertheless, low energy availability was not specifically assessed in the included studies.

### Potential Relevance of Additional Bone Biomarkers

Our analysis primarily focused on osteocalcin and bone alkaline phosphatase, as these were the only markers for which sufficient statistically analyzable data were available. Other bone biomarkers, such as deoxypyridinoline (DPD), pyridinoline (PYD), carboxy-terminal type-1 collagen crosslinks (CTX) and type-1 amino-terminal polypeptide have also been investigated in relation to bone metabolism in some of the studies we included in our current meta-analysis [11, 28, 35, 42, 45, 46]. However, the available data for these markers were limited, heterogeneous, or not directly comparable across studies, which excluded their inclusion in the present statistical analysis. Although these additional biomarkers could not be incorporated into our present meta-analysis, their possible importance should be acknowledged, and the lack of data represents a clear gap in the literature.

### Research and Clinical Implications

We conclude that further research is needed to explore the underlying mechanism of high intensity training, especially in comparison with other training types. Moreover, more studies with standardized exercise protocols are needed to make results more comparable. Physiotherapists, clinicians, and healthcare professionals are encouraged to incorporate high-intensity training as an important tool into exercise plans.

## Conclusion

Here we demonstrate that only high-intensity systematic training significantly affects osteocalcin levels and body mass index, whereas other exercise types such as moderate-to-high, low-intensity, power, and mixed training do not. We also found that osteocalcin synthesis did not depend on duration or frequency, but rather on intensity. Specifically, we observed significant changes in osteocalcin levels exclusively with high-intensity training, regardless of whether the training was of short or long duration. Our analysis revealed a similar pattern in the effect of training on bone-alkaline phosphatase levels. Moderate-to-high-intensity continuous running and endurance training showed a significant decrease in Body Mass Index studies and low-intensity continuous endurance training had a very strong effect on body composition without affecting Bone Mineral Density. Our findings highlight that when the synergistic effect of high-intensity, dynamic loading, and rest periods during training were simultaneously present during systematic training, we observed significant changes in bone metabolism markers and a strong positive trend in the improvement of Body Mass Index and Bone Mineral Density. Our present results indicate that osteocalcin responses are associated with impact-based, high-intensity . interval-type exercise, but further research is needed to determine whether similar effects occur with non-impact training modalities. There is an unmet need for guidelines that uniformly define additional characteristics of training interventions, such as short vs. long duration or high vs. low frequency, to allow for greater consistency and comparability across studies.

## Supplementary Information

Additional file 1.

Supplementary Material 2

## Data Availability

The datasets used in this study can be found in full-text articles included in the systematic review and meta-analysis. All data supporting the findings of this study are available within the paper and its Supplementary Information.

## Abbreviations

BAP: Bone-alkaline phosphatase
BMD: Bone mineral density
BMI: Body mass index
CI: Confidence interval
EN: Endurance training
HF: High frequency
HI: High intensity
HR: Max maximum heart rate
LF: Low frequency
LI: Low intensity
M: Mixed training
MD: Mean difference
OC: Osteocalcin
P: Power training
RCT: Randomized controlled trial
SMD: Standardized mean difference
