# Supplementary material for: High-Intensity Training Increases Osteocalcin Levels: A Meta-Analysis of Effects of Exercise on Bone Remodeling biomarkers
Source: Sports Med Open. 2026 Jun 2;12:60. doi: 10.1186/s40798-026-00986-2 (PMC13230329; doi:10.1186/s40798-026-00986-2)
Supplement: Supplementary file 1 — Additional file 1. [file 40798_2026_986_MOESM1_ESM.docx]

**Title**

**High-Intensity Training Increases Osteocalcin Levels: A Meta-Analysis of Effects of**

**Exercise on Bone Remodeling Biomarkers**

**Short title: Effect of Exercise on Bone Remodeling: Meta-Analysis**

**Viktória Barna^1,2,3,8^, Amir Makolli^1,2,3^, Zsuzsanna Pásztorné Benyó^1,2,3^, Marie Anne Engh^1^, Ádám Zolcsák^4^, Brigitta Teutsch^1,5,6^ Vivienne Seymour^2^, Péter Hegyi^1,6,7^, Renáta Papp^2,3^ †Nora Sydo^9^ †Péter Ferdinandy^,2,3,8^**

**Affiliations:**

1. Centre for Translational Medicine, Semmelweis University, Budapest, Hungary
2. Department of Pharmacology and Pharmacotherapy, Semmelweis University, Budapest, Hungary
3. Center for Pharmacology and Drug Research & Development, Semmelweis University, Budapest, Hungary
4. Department of Biophysics and Radiation Biology, Semmelweis University, Budapest, Hungary.
5. Department of Radiology, Medical Imaging Centre, Semmelweis University, Korányi Sándor U. 2, 1082, Budapest, Hungary.
6. Institute for Translational Medicine, Medical School, University of Pécs, Pécs, Hungary
7. Institute of Pancreatic Diseases, Semmelweis University, Budapest, Hungary
8. Pharmahungary Group, Szeged, Hungary.
9. Heart and Vascular Center, Semmelweis University, Budapest, Hungary

†Equal contribution and last authorship

**Corresponding author**

Péter Ferdinandy

Department of Pharmacology and Pharmacotherapy, Semmelweis University, Budapest, Hungary

Center for Pharmacology and Drug Development, Semmelweis University, Budapest, Hungary

Pharmahungary Group, Szeged, Hungary

**Correspondence**

Péter Ferdinandy, Department of Pharmacology and Pharmacotherapy, Semmelweis University, Budapest, Hungary.

Email: [peter.ferdinandy@pharmahungary.com](mailto:peter.ferdinandy@pharmahungary.com)

**E-mail addresses**

Viktória Barna: [barna.viktoria2@semmelweis.hu](mailto:barna.viktoria2@semmelweis.hu)

Amir Makolli: [amir.makolli@phd.semmelweis.hu](mailto:amir.makolli@phd.semmelweis.hu)

Pásztorné Benyó Zsuzsanna: [pasztorne.zsuzsanna@stud.semmelweis.hu](mailto:pasztorne.zsuzsanna@stud.semmelweis.hu)

Engh Marie Anne: [engh.marie@semmelweis.hu](mailto:engh.marie@semmelweis.hu)

Zolcsák Ádám: [zolcsak.adam@semmelweis.hu](mailto:zolcsak.adam@semmelweis.hu)

Teutsch Brigitta: [teutsch.brigitta1@semmelweis.hu](mailto:teutsch.brigitta1@semmelweis.hu)

Vivienne Seymour: [vivienne.seymour@stud.semmelweis.hu](mailto:vivienne.seymour@stud.semmelweis.hu)

Péter Hegyi: [hegyi2009@gmail.com](mailto:hegyi2009@gmail.com)

Papp Renáta Emese: [papp.renata@semmelweis.hu](mailto:papp.renata@semmelweis.hu)

Nóra Sydó: [nora.sydo@gmail.com](mailto:nora.sydo@gmail.com)

Péter Ferdinandy: [peter.ferdinandy@pharmahungary.com](mailto:peter.ferdinandy@pharmahungary.com)

**Corresponding author**

Péter Ferdinandy M.D., Ph.D.

Department of Pharmacology and Pharmacotherapy, Semmelweis University, Budapest, Hungary

Center for Pharmacology and Drug Development, Semmelweis University, Budapest, Hungary

Pharmahungary Group, Szeged, Hungary

**Correspondence**

Péter Ferdinandy, Department of Pharmacology and Pharmacotherapy, Semmelweis University, Budapest, Hungary.

Email: [peter.ferdinandy@pharmahungary.com](mailto:peter.ferdinandy@pharmahungary.com)

**Supplementary Materials – Index**

**Table of Contents**

[**Additional File 1 Supplementary Methods S1 Prospero registration** 4](#_Toc219220829)

[**Additional File 1 Supplementary Methods S2 Search key and number of hits** 4](#_Toc219220830)

[**Additional File 1 Supplementary Methods S3 - Subcategorization** 5](#_Toc219220831)

[**Additional File 2 Supplementary Table S1 PRISMA-S** 6](#_Toc219220832)

[**Additional File 2 Supplementary Table S2 Result of Grade Assessment** 7](#_Toc219220833)

[**Additional File 2 Supplementary Table S3 Result of Risk of Bias Assessment** 10](#_Toc219220834)

[**Additional File 3 Supplementary Material Figure S1 Overall analysis: Effect of endurance training on osteocalcin levels** 11](#_Toc219220835)

[**Additional File 3 Supplementary Material Figure S2 Effect of long and short duration endurance training on osteocalcin levels** 11](#_Toc219220836)

[**Additional File 3 Supplementary Material Figure S3 Effect of high frequency endurance training on osteocalcin levels** 13](#_Toc219220837)

[**Additional File 3 Supplementary Material Figure S4 Effect of power training on osteocalcin levels** 13](#_Toc219220838)

[**Additional File 3 Supplementary Material Figure S5 Effect of high intensity power training on osteocalcin levels** 14](#_Toc219220839)

[**Additional File 3 Supplementary Material Figure S6 Effect of mixed training on osteocalcin levels** 14](#_Toc219220840)

[**Additional File 3 Supplementary Material Figure S7 Overall effect of endurance training on Bone Mineral Density** 15](#_Toc219220841)

[**Additional File 3 Supplementary Material Figure S8 Effect of high-frequency and high-intensity endurance training on Bone Mineral Density** 16](#_Toc219220842)

# **Additional File 1 Supplementary Methods S1 Prospero registration**

We submitted our study protocol to the International Prospective Register of Systematic Reviews: PROSPERO: CRD42023483811

<https://www.crd.york.ac.uk/prospero/display_record.php?RecordID=483811>

<https://www.crd.york.ac.uk/prospero/display_record.php?ID=CRD42023483811>

# **Additional File 1 Supplementary Methods S2 Search key and number of hits**

**Mediline (via Pubmed)**

**Specification: no filters, all fields**

(Biomarker* OR ("bone" AND "alkaline phosphatase") OR "osteocalcin" OR “pyridinoline”) AND (((„endurance" OR „resistance" OR „strength„ OR „power" OR “aerobic” OR “anaerobic” OR “low intensity” OR “high intensity”) AND train*) OR „exercise" OR „sports") AND random*

**Number of hits: 5 399**

**Embase**

**Specification: all fields, mapping disabled**

(Biomarker* OR (bone AND alkaline phosphatase) OR osteocalcin OR pyridinoline) AND (((endurance OR resistance OR strength OR power OR aerobic OR anaerobic OR low intensity OR high intensity) AND train*) OR exercise OR sports) AND random*

**Number of hits: 6 381**

**Cochrane Central library**

**Specification: all texts**

(Biomarker* OR (bone AND alkaline phosphatase) OR osteocalcin OR pyridinoline) AND (((endurance OR resistance OR strength OR power OR aerobic OR anaerobic OR low intensity OR high intensity) AND train*) OR exercise OR sports) AND random*

**Number of hits: 4 654**

# **Additional File 1 Supplementary Methods S3 - Subcategorization**

Subcategorization of interventions by duration, frequency and intensity.

**Duration of the intervention**

Long-duration intervention training was determined as a controlled, pre/post-measured training activity lasting for more than 16 weeks. Below 16 weeks, we considered the intervention as a short-term regular training.

**Types of exercise interventions**

We used the training classification and abbreviated training types as endurance, power and mixed class**.**

**Characteristics of exercise interventions**

We further classified endurance training according to the metabolic relationship between interventions (aerobic endurance vs. anaerobic endurance exercise). Power training was classified according to muscle type contraction and maximum repetition (1-RM). Training types including both elements were classified as mixed training.

**Frequency, intensity and duration of exercises**

We made subgroups for the different training types by frequency, intensity, and duration. For resistance training, the categorization is based on the intensity of muscle contractions and 1-RM. In resistance training studies, 1-RM refers to "one repetition maximum” used in resistance exercise to determine the maximum power of an individual for a specific exercise. A person can lift the maximum weight for only one exercise repetition. Subjects in the low-intensity power training group in the study performed exercises with an intensity of 80%, focusing on the eccentric phase of the movements. Subjects in the high-intensity power training group (HRT) in the study performed exercises with an intensity of 125% to induce supramaximal eccentric contractions in a controlled manner.

# **Additional File 2 Supplementary Table S1 PRISMA-S**

PRISMA-S: An Extension to the PRISMA Statement for Reporting Literature Searches in Systematic Reviews. Rethlefsen ML, Kirtley S, Waffenschmidt S, Ayala AP, Moher D, Page MJ, Koffel JB, PRISMA-S Group. Last updated February 27, 2020.


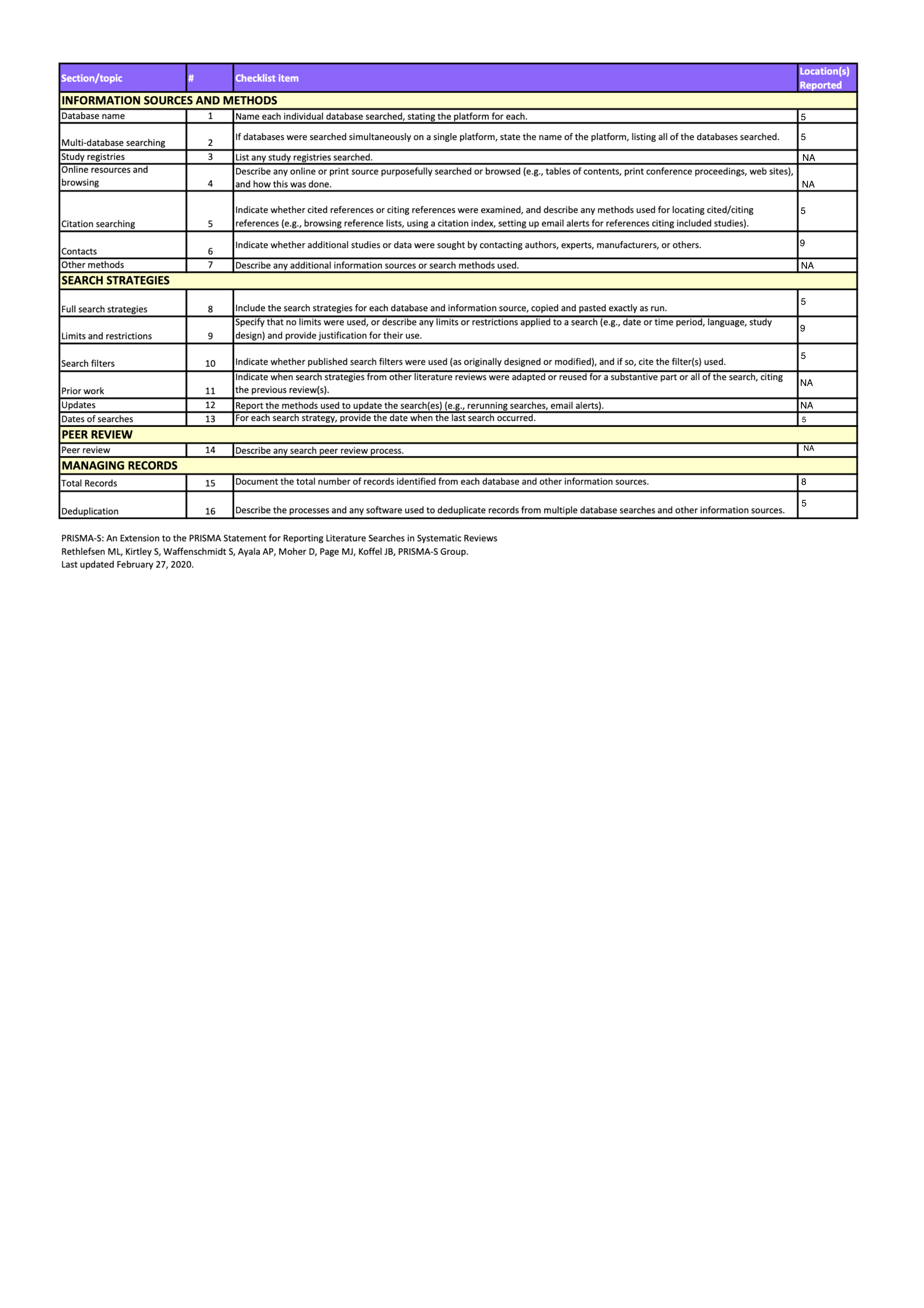


# **Additional File 2 Supplementary Table S2 Result of Grade Assessment**

Summary of findings and quality assessment of the outcomes from Meta-Analysis on the Effects of Different Training Modalities on Osteocalcin (OC), Body Mass Index (BMI), Bone Mineral Density (BMD), and Bone Alkaline Phosphatase (BAP) Levels.

Data presented include anticipated absolute effects with 95% confidence intervals (CI), the number of participants, and the certainty of evidence assessed using the GRADE approach. Abbreviations: MD – Mean Difference, RCT – Randomized Controlled Trial, GRADE – Grading of Recommendations, Assessment, Development, and Evaluation, OC – Osteocalcin, HIIT – High-Intensity Interval Training, BMI – Body Mass Index, BMD – Bone Mineral Density, BAP – Bone Alkaline Phosphatase.

| **Regular training compared to no training in change in osteocalcin, body mass index and bone mineral density** | | | | | |
| --- | --- | --- | --- | --- | --- |
| **Patient or population:** change in osteocalcin, body mass index and bone mineral density  **Setting:**  **Intervention:** regular training  **Comparison:** no training | | | | | |
| **Outcomes** | **№ of participants (studies) Follow-up** | **Certainty of the evidence (GRADE)** | **Relative effect (95% CI)** | **Anticipated absolute effects** | |
|  |  |  |  | **Risk with no training** | **Risk difference with regular training** |
| Osteocalcin Moderate to High intensity training (OC Moderate to High intensity) assessed with: regular training and no training follow-up: range 10 weeks to 16 weeks | 246 (6 RCTs) | ⨁⨁⨁⨁ High^a^ | - |  | MD **95 MD higher** (1.47 lower to 1.33 higher) |
| Osteocalcin in High intensity training group (OC High intensity) assessed with: regular training and no training follow-up: range 12 weeks to 12 months | 193 (6 RCTs) | ⨁⨁⨁⨁ High^a^ | - |  | MD **95 higher** (8.57 higher to 15 higher) |
| Osteocalcin in low intensity training group (OC low intensity ) assessed with: regular training and no training follow-up: range 16 weeks to 12 months | 182 (2 RCTs) | ⨁⨁⨁⨁ High^b^ | - |  | MD **95 higher** (11.51 lower to 9.04 higher) |
| Body Mass Index (BMI) assessed with: regular training and no training Scale from: 18.5 to 30 follow-up: range 10 weeks to 16 weeks | 150 (5 RCTs) | ⨁⨁⨁⨁ High^c^ | - |  | MD **95 higher** (2.07 lower to 0.16 higher) |
| Bone Mineral Density Aerobic Training Group (BMD Aerobic Training) assessed with: regular training and no training follow-up: range 16 weeks to 12 months | 195 (6 RCTs) | ⨁⨁⨁◯ Moderate^a^ | - |  | MD **95 higher** (0.04 lower to 0.3 higher) |
| Bone-alkaline phosphatase (BAP) assessed with: regular training and no training follow-up: range 2 months to 7 months | 208 (5 RCTs) | ⨁⨁⨁◯ Moderate | - | - | SMD **95 SD higher** (0.47 lower to 1.07 higher) |
| ***The risk in the intervention group** (and its 95% confidence interval) is based on the assumed risk in the comparison group and the **relative effect** of the intervention (and its 95% CI).  **CI:** confidence interval; **MD:** mean difference; **SMD:** standardised mean difference | | | | | |
| **GRADE Working Group grades of evidence** **High certainty:** we are very confident that the true effect lies close to that of the estimate of the effect. **Moderate certainty:** we are moderately confident in the effect estimate: the true effect is likely to be close to the estimate of the effect, but there is a possibility that it is substantially different. **Low certainty:** our confidence in the effect estimate is limited: the true effect may be substantially different from the estimate of the effect. **Very low certainty:** we have very little confidence in the effect estimate: the true effect is likely to be substantially different from the estimate of effect. | | | | | |

# **Additional File 2 Supplementary Table S3 Result of Risk of Bias Assessment**


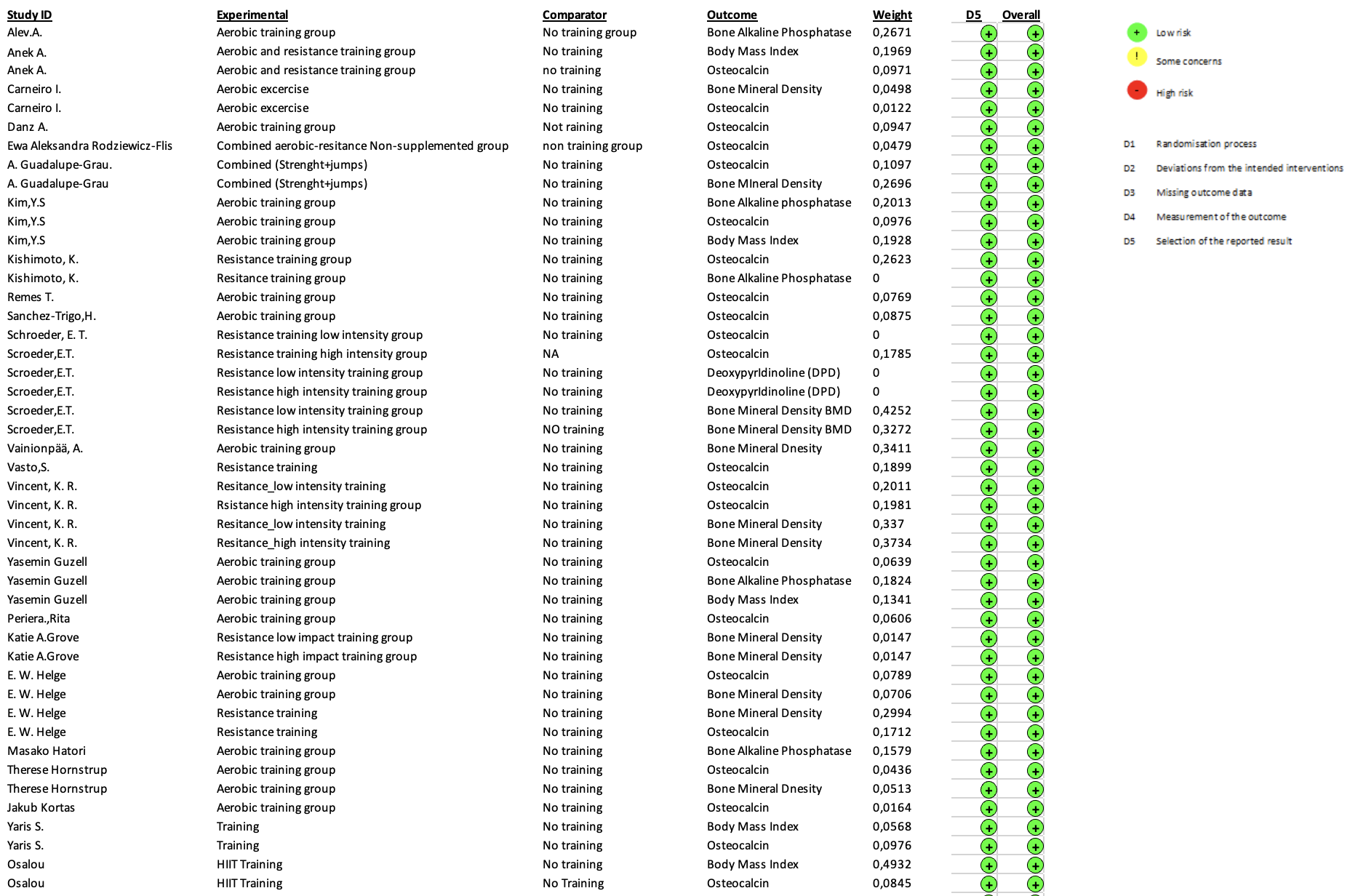


# **Additional File 3 Supplementary Material Figure S1 Overall analysis: Effect of endurance training on osteocalcin levels**


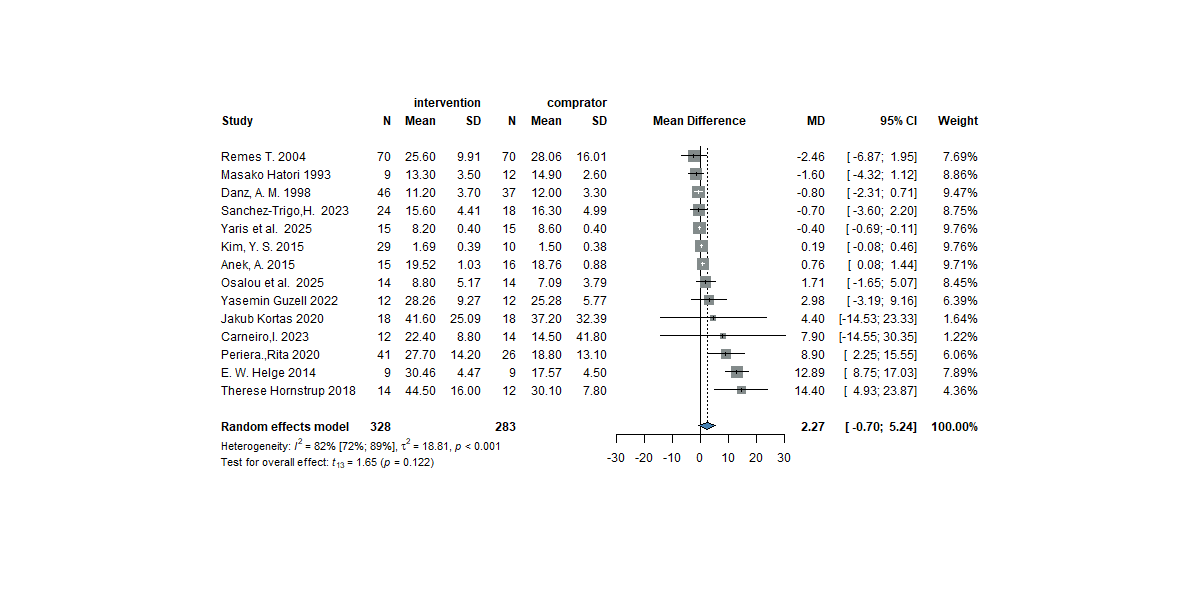


# **Additional File 3 Supplementary Material Figure S2 Effect of long and short duration endurance training on osteocalcin levels**


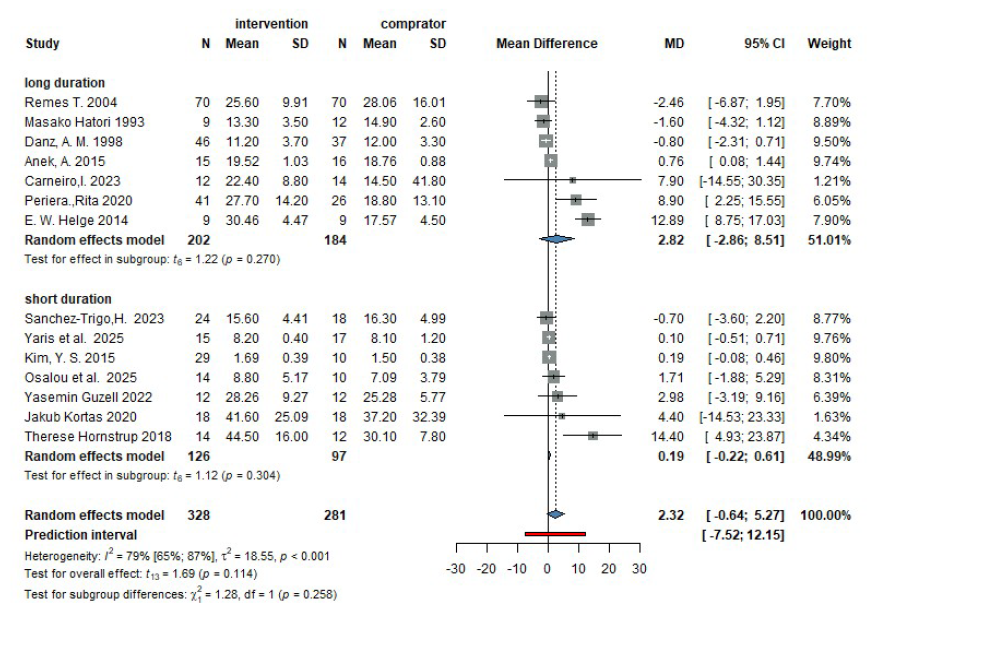


# **Additional File 3 Supplementary Material Figure S3 Effect of high frequency endurance training on osteocalcin levels**


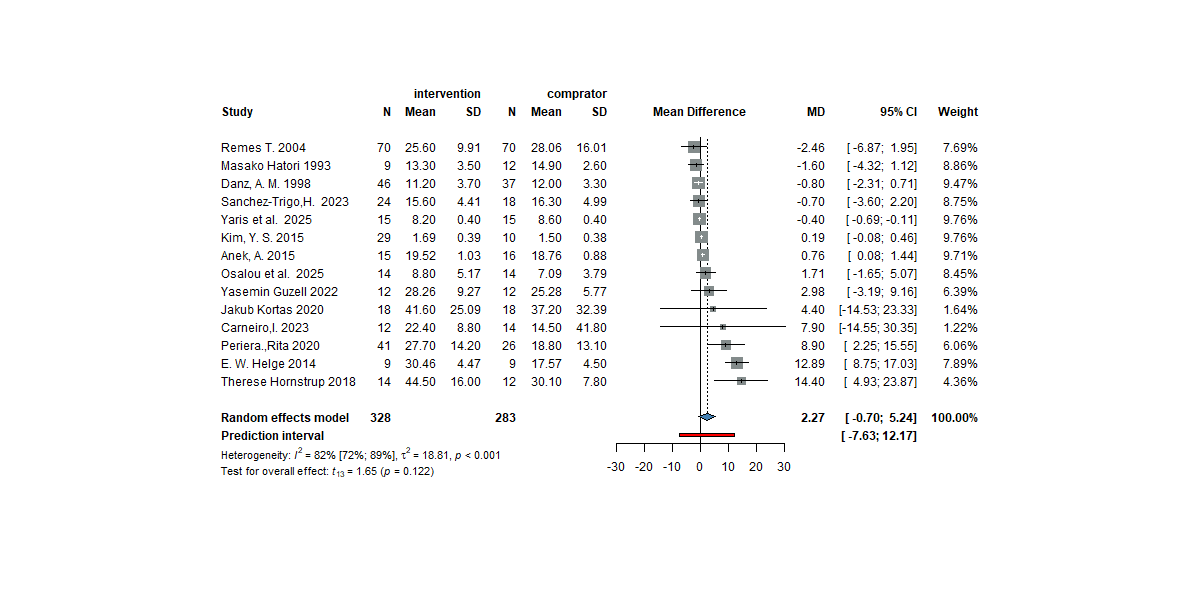


# **Additional File 3 Supplementary Material Figure S4 Effect of power training on osteocalcin levels**


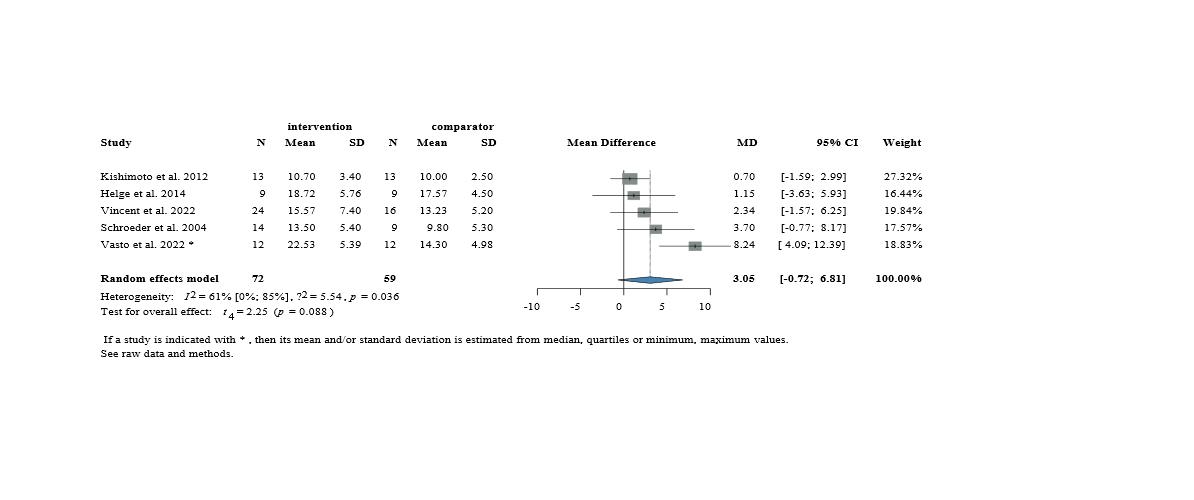


# **Additional File 3 Supplementary Material Figure S5 Effect of high intensity power training on osteocalcin levels**


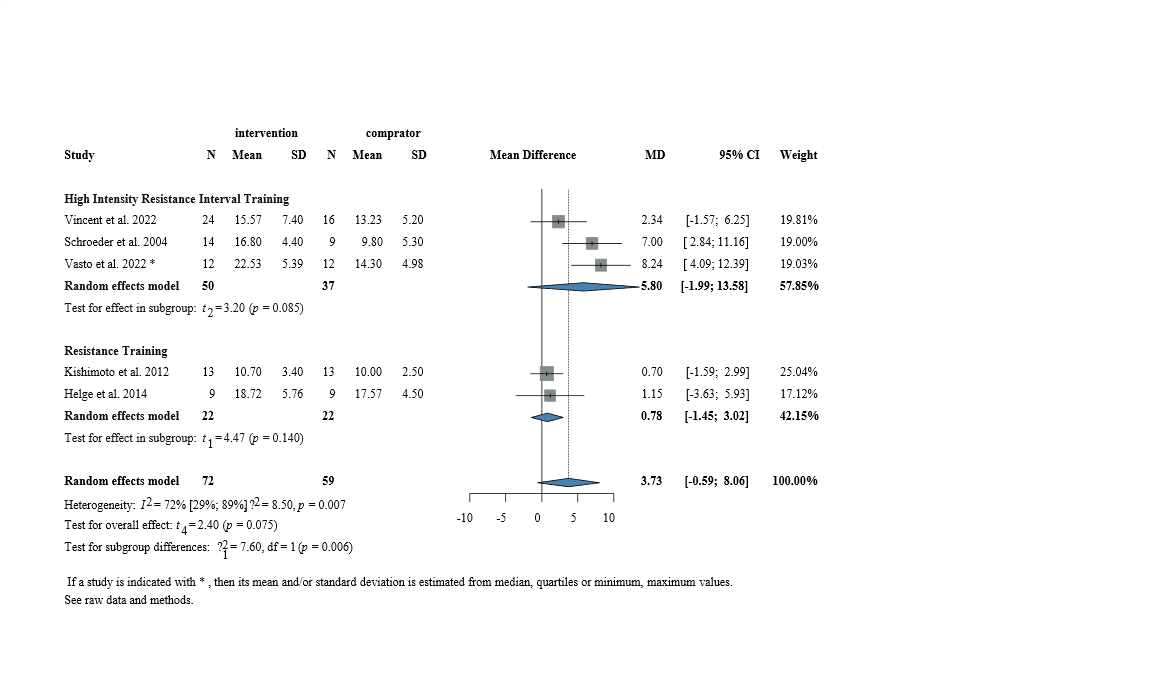


# **Additional File 3 Supplementary Material Figure S6 Effect of mixed training on osteocalcin levels**


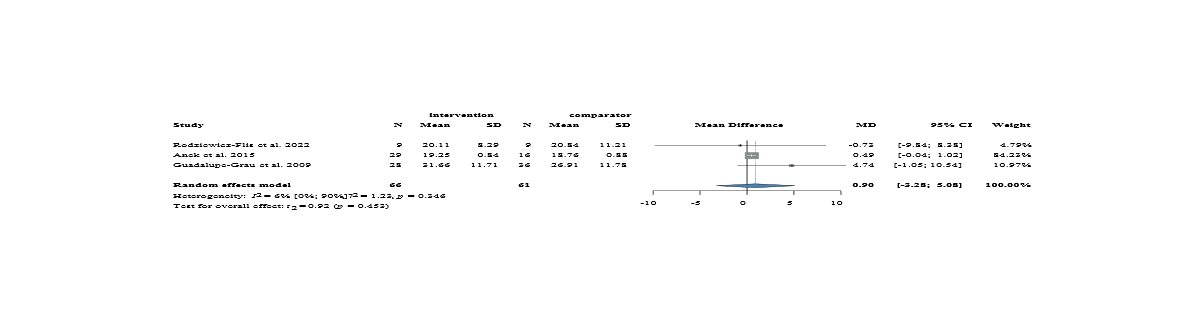


# **Additional File 3 Supplementary Material Figure S7 Overall effect of endurance training on Bone Mineral Density**

# **Additional File 3 Supplementary Material Figure S8 Effect of high-frequency and high-intensity endurance training on Bone Mineral Density**

**
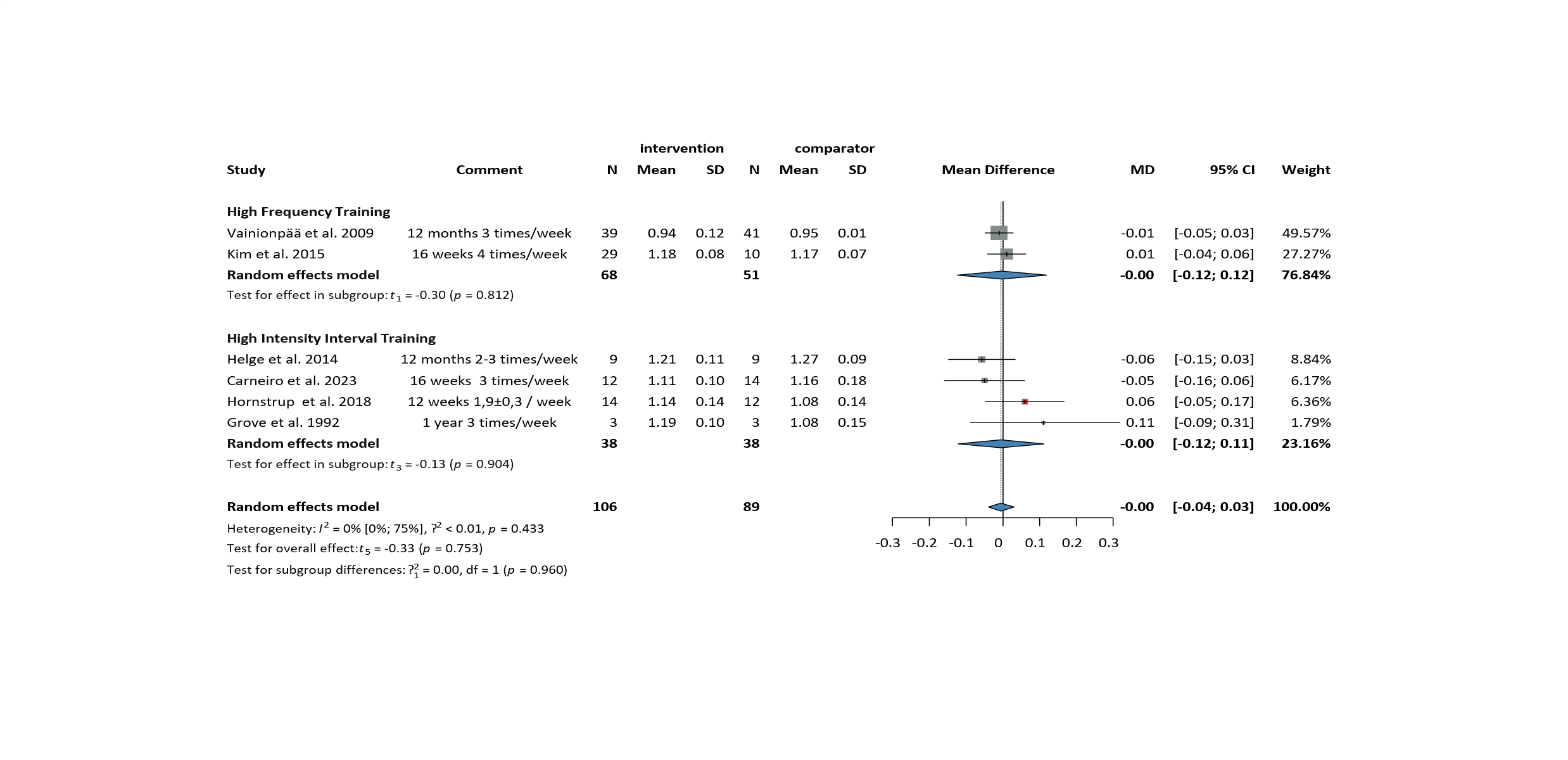
**
